# Factors influencing sex ratio at birth in Krosno, Poland

**DOI:** 10.1038/s41598-023-50555-w

**Published:** 2024-01-02

**Authors:** Joanna Nieczuja-Dwojacka, Justyna Marchewka-Długońska, Alicja Budnik, Patryk Wojtowicz, Bogdan Giemza, Bożena Skrzypczyk, Aneta Zvarik

**Affiliations:** 1 Institute of Biological Sciences, Faculty of Biology and Environmental Sciences, Cardinal Stefan Wyszynski University in Warsaw, 01-938 Warsaw, Poland; 2 John Paul II Podkarpackie Province Hospital in Krosno, 38-400 Krosno, Poland

**Keywords:** Paediatric research, Outcomes research

## Abstract

The secondary sex ratio (SSR) is a widely used descriptor that reflects the living conditions and health status during pregnancy. The aim of study was to assess the impact of maternal factors, season of birth, and air pollution with the heating season on the sex ratio at birth in the Subcarpathian population from the Krosno district, Poland. A retrospective study involving 11,587 births was occurred at the John Paul II Podkarpackie Province Hospital in Krosno between 2016 and 2020. Sex of the newborn, the season of their birth, as well as the maternal age, birth order, the interval between births, and the season of birth were analysed. Furthermore, the relationship between the SSR and the level of air pollution during the heating season was investigated. To determine the significance of differences in sex ratios, chi-square analysis and multifactorial regression were used, with a significance level set at *p* < 0.05. At the chi-square level, all the studied factors indicated a statistically significant relationship with the SSR. However, the regression model used shows that maternal age and birth order were the most important factors in shaping the SSR in the study group.

## Introduction

The secondary sex ratio (SSR), also referred to as the sex ratio at birth, is a measure that quantifies the variation in the sex ratio of newborns^1–3^. The SSR is calculated by dividing the number of male live births by the total number of births during a specific period^1^, or by expressing the number of live male births per 100 females^4^. In Western countries, the SSR ranges from 1.04 to 1.09^5,6^.

Irrespective of the method used to estimate the SSR, it is recognized that it can be used as a reflection of living conditions and health status. It is assumed that deteriorating conditions and declining health are associated with a decrease in SSR values while improving conditions are linked to an increase, although not all data support this phenomenon. Furthermore, the mechanisms for this sex disparity are not well-defined^7–11^.

The term “secondary sex ratio” is intended to distinguish it from the primary sex ratio (PSR), which represents the sex ratio at conception. In human populations, the PSR is expected to be 1:1 based on the law of independent assortment of the X and Y chromosomes^12^. However, measuring the PSR at conception is challenging, leading to limited knowledge in this area^13^. Scientific literature often suggests that the PSR is more male-biased than the birth sex ratio^14,15^. Nowadays, the claim of higher male mortality during pregnancy is often advanced as part of a claim of life-long male fragility^16^. Estimates of the PSR in these studies typically indicate a proportion of males at 0.56 or greater. Some researchers have concluded that the PSR is unbiased or slightly male-biased^17,18^. However, as Orzack et al.^19^ point out, there is insufficient evidence to support such estimates. A male-biased PSR aligns with a male bias in spontaneous abortions and the sex ratio at birth, but an unbiased or even female-biased sex ratio is also possible depending on early pregnancy dynamics^20^.

Despite some reservations, the SSR is widely used in research as a measure of exposure to stressors during pregnancy^10,12^. Numerous studies have consistently shown an excess of male newborns among live births, which holds true for historical data as well^21–23^. Researchers have explored various factors to explain the higher number of male newborns, including socio-economic status and parental education^24^, parental and maternal age^25,26^, birth order^27^, the season of birth^28^, solar radiation intensity^29^, preconception stress levels and stress during pregnancy^30,31^, and smoking and environmental pollution levels^32–35^. However, it should be noted that while these studies provide insights into the potential causes of gender differences at birth, the results are often inconclusive. Thus, it can be assumed that the SSR is the result of multiple factors that interact simultaneously during pregnancy.

The aim of this study was to assess the impact of the mother’s age, birth order, interpregnancy intervals, and the season of birth on the SSR coefficient, while also considering the influence of air pollution during the heating period. Based on existing literature, the research hypotheses propose that better environmental conditions, including maternal factors, are associated with a higher likelihood of male births. It is anticipated that the highest number of male births will occur when women are in their optimal biological age range of approximately 25–30 years. Regarding the order of pregnancies, it is hypothesized that the second or third pregnancy, which is also correlated with the mother’s age, will be most conducive to the development of male fetuses. Most researchers concur that an interpregnancy interval of around 2 years is most favorable, suggesting a greater likelihood of male deliveries within such a timeframe. Additionally, male newborns are expected to be more prevalent during the winter/spring season. These births are conceived during the summer or early autumn when there is ample exposure to vitamin D and a diverse range of seasonal foods. However, it should be noted that the winter season in Poland coincides with increased air pollution due to the heating season^36^. This air pollution can have detrimental effects on the developing fetus and potentially influence changes in the SSR^37–41^.

## Methods

The retrospective cross-sectional study material consisted of data on newborns and their mothers, collected at the John Paul II Podkarpackie Province Hospital in Krosno in the years 2016–2020. The data was obtained with the approval of the Ethics Committee of the John Paul II Podkarpackie Province Hospital in Krosno. Informed consent was obtained from all subjects and/or their legal guardian(s).

The county is located within the Carpathian Foothills, Jasielsko-Krośnieńska Valley and Low Beskids, in the southeast of Poland. It is characterized by one of the lowest urbanization rates. The number of inhabitants of the Krosno district in 2021 was 112,195 people. In the entire Podkarpackie Voivodeship, a higher proportion of boys were recorded among live births, 51.9%, which gives a slightly higher share of this sex in relation to the data from the whole country (51.4%). On average, fewer children were born in Krosno compared to other districts of the Podkarpackie Voivodeship, with rates of 7.7/1,000 inhabitants and 10/1,000 inhabitants. In the case of the fertility rate, defined as the average number of live births/1,000 women of reproductive age, Krosno recorded its lowest value compared to other districts of the voivodship (Krosno 31.7, average for the voivodeship 40.5). In the Podkarpackie Voivodship, a shift of the highest fertility to older age groups was observed: in 2019, there were 38 live births/1,000 women in the 20–24 age group, 93 births in the 25–29 age group, and 84 births in the 30–34 age group^42,43^.

A total of 11,587 cases were included in the analysis, which consisted of both singleton and twin pregnancies. There were no information available in the database about identical/fraternal twin births. However, the SSR values for twin pregnancies were examined separately and subsequently excluded from further analyses. Out of the total cases, 98 were twin pregnancies, resulting in 49 deliveries.

The age of the women ranged from 15.2 to 49.2 years, with an average age of 30.2 years. Among the studied women, 39.4% were primiparas (first-time mothers), 39.2% had previously given birth once, and the remaining 21.3% had three or more prior childbirths. Because gender identity was not a subject of this study, only biological sex was examined, and it is biological sex, not gender identity, that the authors refer to when using the term “pregnant women”.

In the dataset, there were a total of 6279 male newborns and 5305 female newborns from singleton deliveries. For twin deliveries, the sex ratio was equal, with 40 males and 49 females. This resulted in a sex ratio of 1:1 for twins and a sex ratio of 1:1.18 (5258:6231) for singleton deliveries. Table 1 provides a summary of the respondents’ ages based on the number of childbirths.

**Table 1:** Age of women’s depending on the number of previous deliveries.

Previous deliveries	Mean (+ / − SD)	Range
None [N = 4378]	27.5 (5.21)	15.1–47.6
1 [N = 4347]	30.8 (4.57)	18.6–46.0
2 [N = 1732]	33.7 (4.40)	19.6–46.6
> 2	34.9 (5.82)	20.5–49.2
Total	30.2 (5.45)	15.2–49.2

Where: N – total number of women depending on the number of previous deliveries.

To examine the influence of the mother’s age on the SSR, the data for singleton births were divided into five age groups, following the categorization by Jacobsen et al.^27^. The age groups were as follows: less than 19.9 years, 20–24.9, 25–29.9, 30–34.9, and over 35 years old. The SSR value was estimated for each age group.

Subsequently, the study investigated the impact of birth order on the secondary sex ratio. The women were divided into four groups based on the order of their current birth: first, second, third, and subsequent births.

The relationship between the SSR and the interval between the current and previous births was also examined. Women were divided into the following categories based on the time elapsed between births: 0–2, 2–4, and 4 or more years.

Furthermore, the study analyzed the relationship between the season of birth and the SSR. Newborns were divided into four groups corresponding to the seasons: spring (March, April, and May), summer (June, July, and August), autumn (September, October, and November), and winter (December, January, and February). The aim was to assess how the SSR varied depending on the seasons during which the trimesters of pregnancy occurred. Additionally, it was important to investigate whether the pregnancies coincided with the so-called heating period when increased emissions from heating gas are observed throughout Poland.

Air pollution data, including variations in concentration over the year for PM10 [µg/mm^3^], PM2.5 [µg/mm^3^], SO2 [µg/m^3^], NOx [µg/m^3^], and PAH (polycyclic aromatic hydrocarbons) [ng/m^3^], were obtained from the Chief Inspectorate of Environmental Protection^44^. Pollution values from measuring stations located in the Krosno district were averaged. Regardless of the registration method (24 h or 1 h) the data collected from the measurement stations were averaged (according to the registration time), and then the results were compared using the ANOVA test (the grouping factor was the year off registration). Since the ANOVA did not show statistically significant differences, it was decided to average the results for individual months in the examined years.

To determine the significance of differences, the chi-square test was employed, while logistic regression was utilized to estimate the odds ratio. Statistical analyses were performed in Statistica 13. 0. First, it was checked whether in the case of a single factor (e.g. heating seasons and the associated increase in air pollution) its impact on the sex ratio could be seen. However, because that this is a multifactorial phenomenon, we decided to use a regression model to show us how the variables we examined collectively affect the sex ratio. So if we only analyze the results for the chi-square test, we can see how a single factor affects the sex ratio. If we put these factors in the model, we can see how all of them together influence the sex ratio and which of them have the greatest impact on this phenomenon.

## Results

### Twin births

The SSR was initially examined for both multiple and single pregnancies. The findings are presented in Table 2. Out of the total study sample, there were 89 recorded twin pregnancies, resulting in an equal sex ratio of 1:1. For singleton pregnancies, the SSR index was determined to be 118.5.

**Table 2:** Sex ratio of twin and singleton births.

Type of birth	Newborn sex n [%]		Sex ratio
	Female	Male	
Twins [N_1_ = 89]	49 [50.0]	49 [50.0]	100.0
Singelton [N_1_ = 11489]	5258 [45.8]	6231 [54.2]	118.5
Total (N = 11578)	1736 [47.8]	1897 [52.2]	118.3

Where: N – total number of newborns; N_1_ – total number of newborns in the type of birth category; n – number of newborns in the sample; calculated as [(n/N_1_)*100] or [(n/N)*100].

### Maternal age

The chi-square test results indicate a statistically significant relationship between the age of the mother and the sex of the newborn. Subsequently, the odds ratio was calculated for each age category. Table 3 shows the data, revealing that the likelihood of having a male newborn increases for women over 30 years of age. Among women aged 30–35, the odds ratio is 1.25, indicating a 25% higher chance of having a male newborn. For women over 35, this chance increases to 35%. When the study group is divided into two categories—those under 30 and those over 30—it is observed that the chance of having a male newborn increases by 15% in women over 30 (OR 1.152; 95% CI 1.06–1.25).

**Table 3:** The sex of the newborn depends on the age of the mother (age categories designated after Jacobsen et al. 1999).

Maternal age [years]	Newborn sex n [%]		Sex ratio	Odds ratio (95% CI)
	Female	Male		
< 19.9 (N = 323)	153 [47.4]	170 [52.6]	111.1	1.001 (0.93–1.09)
20.0–24.9 (N = 1651)	780 [47.2]	871 [52.8]	111.7	1.163 (0.97–1.52)
25.0–29.9 (N = 3604)	1721 [47.8]	1883 [52.2]	109.4	1
30.0–34.9 (N = 4220)	1904 [45.1]	2316 [54.9]	121.6	1.256 (0.84–1.88)
> 35.0 (N = 1688)	698 [41.3]	990 [58.6]	141.8	1.349 (1.08–1.68)
Chi-square test		*p* = 0.0002 (χ = 21.48; df = 4)		

Where: N – total number of newborns born to women of a given age; n – number of female or male newborns depending on the age of the mother; % calculated as [(n/N)*100].

### Birth order

The chi-square test showed a statistically significant relationship between the order of delivery and the sex of newborns. Table 4 displays the data, indicating that the SSR value for first pregnancies is 119.2. However, for second pregnancies, the SSR decreases to 109.2, and then it increases to 130.9 for newborns born from third pregnancies. The odds ratio was used to estimate the probability of having a male newborn in different pregnancy orders. It was found that compared to the first pregnancy, the likelihood of having a male newborn increased by 20% in the third pregnancy. For each subsequent pregnancy, the increase in the probability of having a male newborn is less than 9% compared to the first pregnancy.

**Table 4:** Secondary sex ratio and birth order.

Birth order	Newborn sex n [%]		Sex ratio	Odds ratio (95% CI)
	Female	Male		
1 (N = 4357)	1988 [45.6]	2369 [54.4]	119.16	1
2 (N = 4269)	2053 [47.8]	2243 [52.2]	109.25	0.887 (0.72–1.105)
3 (N = 1716)	743 [43.3]	973 [56.7]	130.95	1.200 (0.91–1.58)
> 3 (N = 596)	260 [43.6]	336 [56.4]	129.23	1.084 (0.91–1.29)
Chi-square test		*p* = 0.0068 (χ = 12.17; df = 3)		

Where: N – total number of newborns depending on birth order; n – number of female or male newborns depending on birth order; % calculated as [(n/N)*100].

### Interpregnancy intervals

Further analysis was conducted to investigate the relationship between interpregnancy intervals and the SSR. The chi-square test was performed, and it revealed a statistically significant association, even after considering 5624 multiparous women. The results displayed in Table 5 demonstrate that the highest SSR value was recorded in pregnancies where the interpregnancy interval exceeded 4 years.

**Table 5:** Time between pregnancies and SSR.

Time between pregnancies [years]	Newborn sex n [%]		Sex ratio	Odds ratio (95% CI)
	Female	Male		
0–1.9 [N = 1199]	564 [47.0]	635 [53.0]	112.59	1.218 (0.95–1.55)
2.0–4.0 [N = 1753]	859 [49.0]	894 [51.0]	104.07	1
> 4 [N = 2672]	1154 [43.2]	1518 [56.8]	131.54	1.264 (1.12–1.42)
Chi-square test		*p* = 0.0005 (χ = 15.32; df = 2)		

Where: N – total number of newborns depending on interval between pregnancies; n – number of female or male newborns depending on interval between pregnancies; % calculated as [(n/N)*100].

Comparing the odds ratio values for the group with interpregnancy intervals of 2–4 years, it can be concluded that each birth occurring over 4 years increases the chance of giving birth to a male newborn by more than 26%. Similarly, in the case of interpregnancy intervals below 2 years, the chance is slightly lower but still amounts to almost 22%.

The time between pregnancies was also examined. Based on these findings, it can be concluded that the shortest interval occurs between the first and second pregnancy, while longer intervals exceeding 4 years are observed between subsequent pregnancies, with the maximum interval occurring between the third and fourth pregnancy.

### Season of birth

The relationship between the season of birth and the sex of the examined newborns was also analyzed. The chi-square test revealed a statistically significant association between these variables. Table 6 presents the results, indicating that the highest SSR value was observed for newborns born in autumn, while the lowest SSR value was recorded for those born in spring.

**Table 6:** Season of the birth and secondary sex ratio.

Season	Newborn sex n [%]		Sex ratio
	Female	Male	
Spring (N = 2809)	1364 [48.6]	1445 [51.4]	105.94
Summer (N = 2932)	1305 [44.5]	1627 [55.5]	124.67
Autumn (N = 2993)	1268 [42.4]	1725 [57.6]	136.04
Winter (N = 2755)	1321 [47.9]	1434 [52.1]	108.55
Chi-square test		*p* = 0.0000 (χ = 29.92; df = 3)	

Where: N – total number of newborns depending on birth season; n – number of female or male newborns depending on birth season; % calculated as [(n/N)*100].

### Heating season

**Figure 1:**
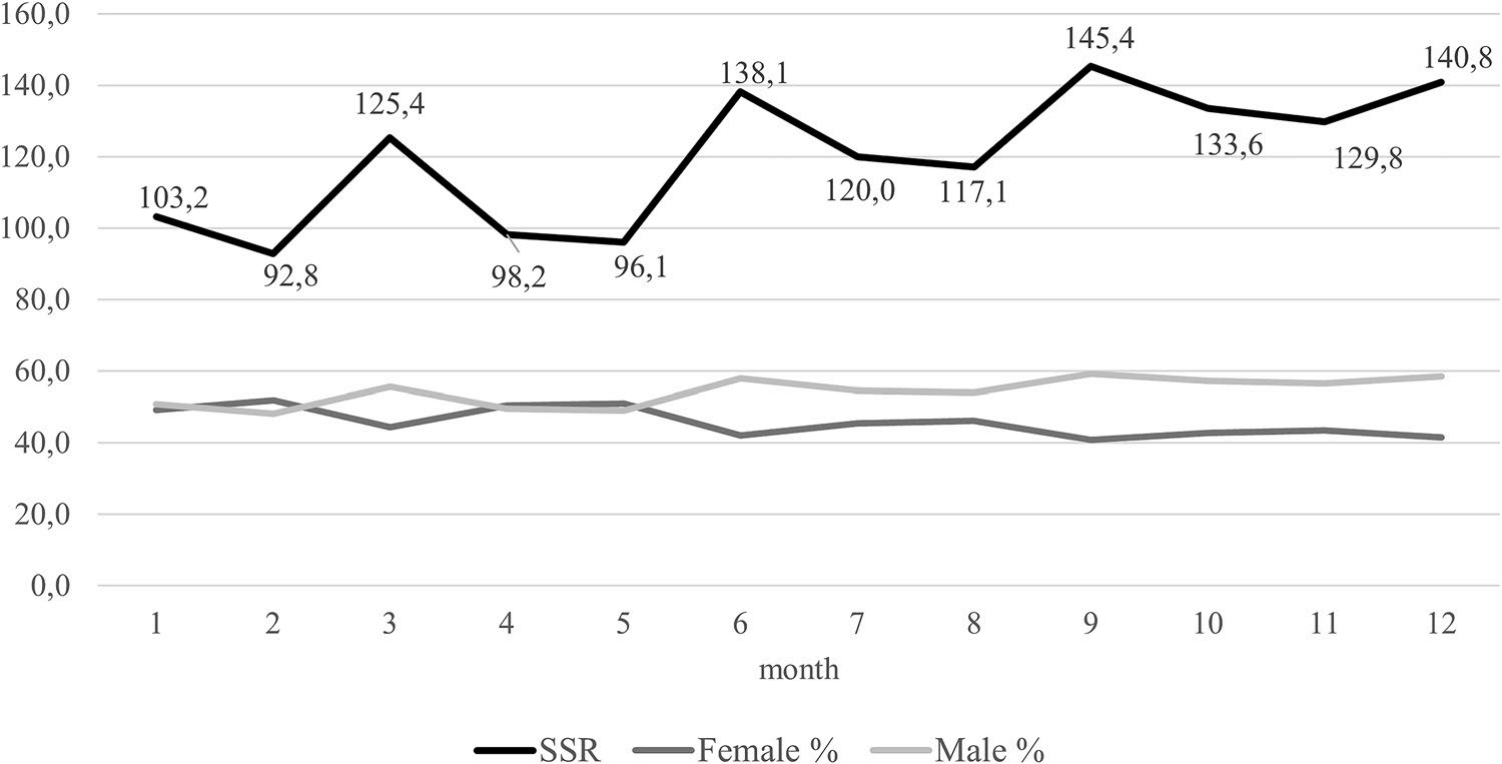
SSR and percentage ratio of boys and girls born in particular months of the year.

The analysis also considered whether there is a relationship between the heating season and the SSR value. In Poland, the heating season typically lasts about 7 months, from September to April. In the study sample from 2016 to 2020 in Krosno, it was observed that during the spring and summer months, nearly 66.34% of all newborns were born. In relation to newborns born between May and September, the SSR value is significantly higher, amounting to 126.5 compared to second season. The chance of giving birth to a male newborn during the months outside the heating season is over 45% higher. These results were statistically significant (Table 7). Figure 1 presents the SSR and the percentage of births for both sexes, highlighting that a significantly higher number of boys are born in the second half of the year.

**Table 7:** Heating season and SSR.

Heating season	Newborn sex n [%]		Sex ratio	Odds ratio (95% CI)
	Female	Male		
Yes (October–April) (N = 3867)	1893 [48.9]	1974 [51.1]	104.28	1
No (May–September) (N = 7622)	3365 [44.2]	4257 [55.8]	126.5	1.45 (1.28–1.45)
Chi-square test		*p* = 0.0000 (χ = 23.85; df = 1)		

Where: N – total number of newborns in born in heating or no heating season; n – number of female or male newborns born in heating and no heating season; % calculated [(n/N)*100].

**Figure 2:**
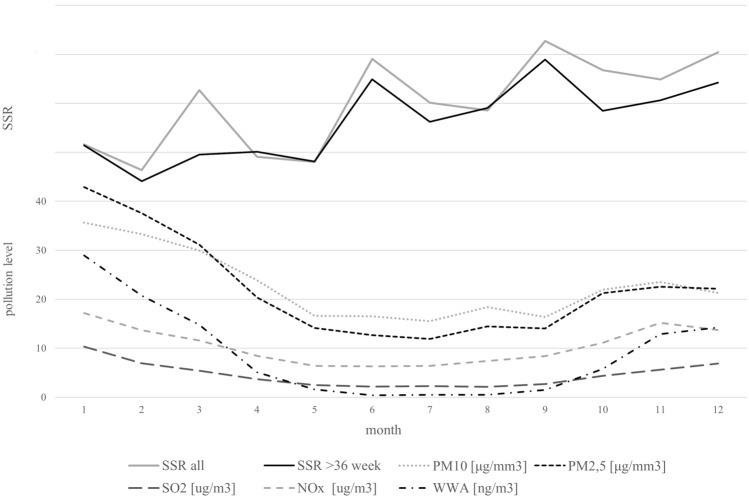
SSR and concentration of PM10 [µg/mm^3^], PM2.5 [µg/mm^3^], SO_2_ [µg/m^3^], NOx [µg/m^3^], and PAH [ng/m^3^].

Additionally, Fig. 2 illustrates changes in SSR and the concentration of pollutants such as PM10 [µg/mm3], PM2.5 [µg/mm3], SO2 [µg/m3], NOx [µg/m3], and PAH [ng/m3] across the year. The figure indicates that fewer boys were born during periods of increased pollutant concentration, which was confirmed by the chi2 test (*p* = 0.0000; χ =23.85; df = 1).

**Figure 3:**
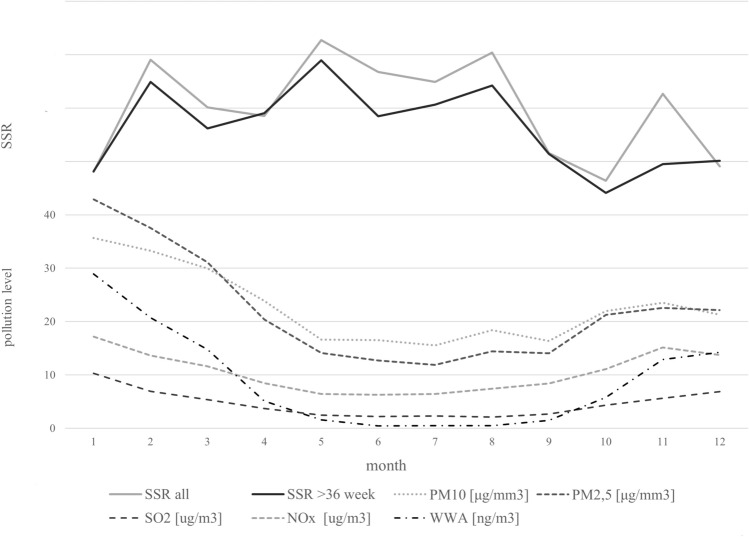
Fertilization month and concentration of PM10 [µg/mm^3^], PM2.5 [µg/mm^3^], SO_2_ [µg/m^3^], NOx [µg/m^3^], and PAH [ng/m^3^]. [1–12—subsequent months].

Figure 3 illustrates changes in SSR in the fertilization period and the concentration of these pollutants across the year. The SSR appears relatively high when the pollutant concentration is low and increases as the concentration of pollutants increases. This suggests that the tested air pollutants may potentially influence the subsequent SSR.

Furthermore, a multifactor regression model was used in the analysis, including all variables except whether the newborn was from a single or twin pregnancy. The results, as summarized in Table 8, indicate that the model used is statistically significant (*R* = 0.2383). When examining the individual components of the model, it becomes apparent that maternal age and birth order have the largest influence on the sex ratio.

**Table 8:** Multifactor regression results.

Factor	Df	MS	*F*	*p*-value
Maternal age	3	1.1960	5.0273	0.0019
Birth order	2	0.9980	4.1951	0.0156
Interpregnancy intervals	3	0.2013	0.8460	0.4691
Season of birth	3	0.4688	1.9706	0.1174
Heating season	1	0.0598	0.2515	0.6163
Full model	12	0.6253	2.6286	0.0020

### Institutional review board statement

The study was conducted in accordance with the Declaration of Helsinki and approved by the Ethics Committee of the John Paul II Podkarpackie Province Hospital in Krosno for studies involving humans.

## Discussion

The most commonly reported value of SSR in research results is between 105 and 107, indicating an excess of male births as a well-documented worldwide phenomenon. Moreover, depending on the factors being examined, the SSR values can significantly vary, emphasizing the male sex predominance. This discrepancy may be attributed to the fact that besides genetic abnormalities that increase the risk of miscarriage, the occurrence of miscarriage itself may be influenced by numerous other factors and their interactions^45–47^. These factors may include physical and psychological health conditions. Sociodemographic factors such as age, occupation, and pre-existing health conditions like high or low body mass index, diabetes, high blood pressure, cancer, infertility, and other diseases or conditions can also contribute to pregnancy loss^48–51^. So if spontaneous miscarriage is defined as the loss of a fetus before the completion of the 20th week of gestation, according to results presented by Orzack et al.^19^, for most of this we should observe a higher probability of miscarriage of female fetuses. However, most studies on miscarriage incidence and risk factors do not record the sex of aborted fetuses, making it difficult to determine how these factors affect the sex ratio. Therefore, further research investigating the influence of these factors on the sex ratio at birth is warranted.

### Twin births

In the study sample, the sex ratio for twin pregnancies was 1:1, while for single pregnancies, the SSR ratio was 118.50. However, it is important to note that the percentage of twin pregnancies in the study sample was very low (0.42%). According to the literature, twin pregnancies typically account for about 1–2% of all pregnancies, and their prevalence has been increasing in recent years^52,53^. This rise in twin pregnancies is believed to be a result of factors such as advanced maternal age at conception and the use of assisted reproductive techniques^54^.

In our sample, the percentage of twins is notably lower, which can be attributed to the characteristics of the hospital where the newborns were delivered. As per Polish regulations, this hospital is classified as a second-degree referral center, which means that high-risk pregnancies, including multiple pregnancies, are typically transferred to a higher-level referral center, such as Rzeszów, located approximately 56 km away^55^. This is particularly relevant for preterm births, as twin pregnancies are at a higher risk of premature delivery, as well as other complications during pregnancy^56–59^.

Therefore, the observed sex ratio value of exactly 1:1 in the study sample may be distorted, considering the lower SSR values commonly observed in multiple pregnancies. Nonetheless, the obtained value is lower than in singleton pregnancies, which is consistent with previous research findings^60^.

### Maternal age

In this study, we observed that mothers under the age of 19, the youngest group, had a lower proportion of male births compared to mothers in other age groups. This could be attributed to the biological immaturity of young women, as male fetuses may require greater metabolic investments from mothers to ensure their survival. The mother’s body may compete for resources with the developing fetus during this stage^61,62^.

Regarding optimal maternal ages, previous studies have reported that younger women tend to give birth to more sons than older women, although not all findings are conclusive^63–66^. Rapaport et al.^67^ observed that for maternal age ≤22, girls were born more often, while the likelihood of having a son increased for middle-aged mothers, with the highest probability occurring at around 31.3 years of age. However, for the oldest women, approaching the perimenopausal period, the probability of having a son decreased again. The authors note that the study population was not affected by urbanization-related factors such as access to contraception, processed food, or air/water pollution. It is worth noting that the women from Krosno County, the population under study, also belong to a traditional population strongly influenced by the doctrine of the Catholic Church. In this group, lower SSR values were observed among younger women, while higher values were noticed among older women above the age of 30. These relationships may also be associated with ongoing social transformations. Currently, it is observed that women are delaying motherhood decisions and placing great importance on completing higher levels of education and pursuing professional careers. Numerous studies also highlight that advanced maternal age trends are linked to poorer newborn outcomes and a higher risk of conditions such as Down syndrome and autism^68,69^. Additionally, it is worth mentioning that research by Sánchez-Barricarte^3^ indicates that the age of mothers, rather than fathers, may have a significant impact on SSR differences.

### Birth order

In this study, a decrease in the SSR value was observed for the mother’s second delivery, while an increase was observed for the third and subsequent pregnancies. This finding differs from the results reported by Jacobsen et al.^27^, where changes in the coefficient value were found to be insignificant. This discrepancy suggests that this factor may not have a significant influence on the secondary sex ratio. Some researchers believe that newborns from earlier pregnancies are more likely to be male, while those from subsequent pregnancies are more likely to be female. However, not all data support this pattern. Furthermore, some researchers have not found a significant association between the number of previous births and the sex ratio^70^.

### Interpregnancy intervals

The lack of a noticeable effect of the number of pregnancies on the SSR may be partly explained by the influence of birth intervals between pregnancies. In this study sample, birth interval data were available for only a portion of the surveyed women (5,330), but the results indicate a relationship between birth intervals and SSR. The impact of birth intervals on the SSR is not well understood. However, in the case of women with higher parity and shorter interdelivery intervals, a higher frequency of daughters being born has been reported. Some scientists attribute this trend to maternal depletion^71^. In certain countries, for social or cultural reasons, women who already have a son may choose to have longer interdelivery intervals compared to women who do not have a male child, which could indirectly affect the SSR^72,73^.

### Season of birth

The work also discusses the influence of environmental factors. In the current study, we found that the highest SSR value was observed for newborns conceived in winter, while the lowest value was recorded for children conceived at the end of summer. The season of conception is believed to play a role in determining the sex of the fetus, with more male fetuses conceived under more favorable environmental conditions. Some researchers have noted that mothers who are pregnant during summer and winter are more likely to give birth to female babies compared to those pregnant in the spring^74^.

The seasonality of SSR is a topic that is still widely debated. It is suggested that the seasonality of human reproduction may be influenced by biological factors related to seasons, insolation, and food availability^75,76^. Research conducted in Germany in the late 1990s indicated that the highest SSR values were observed in May and December, while the lowest values were observed in March and October. This suggests that pregnancies conceived in late summer and spring tend to result in more male births, while those conceived in early summer and winter tend to result in more female births. One possible explanation for this phenomenon is the better reproductive success of men born in the spring compared to those born in the autumn^77^. It is assumed that this may be related to slightly worse conditions of fetal development due to the season of illness, for example flu or vulnerability to vitamin D deficiency^78^.

When considering the seasonality of births, it is also worth mentioning the availability of vitamin D and insolation. Some authors propose a positive relationship between SSR values and maternal vitamin D levels before conception. Optimal levels of vitamin D are believed to reduce inflammation, which could be detrimental to male embryos^79^. Reports from the USA also indicate that higher solar radiation is associated with a higher SSR compared to regions with lower solar radiation. Researchers compared SSR data from southern states with higher insolation to northern states, and significant differences were observed^3^.

### Heating season

In Poland, the SSR may also be influenced by pollution levels during the heating season. In this study, we observed that newborns conceived during the winter season and early spring had a higher SSR compared to those conceived outside the heating season. The impact of pollution on the SSR is not fully understood, and research results are varied. Some studies exclude the influence of pollution on the SSR^3^, while others suggest that pollution, particularly water pollution, can decrease the SSR value^40^. Other reports indicate that maternal exposure to dioxins, pesticides, and lead may result in lower SSR values, and inhalation of particulate matter may also affect the SSR^80–82^. It is worth noting that in many developed countries, the SSR has decreased over the past five decades, and experts attribute this decline to maternal exposure to pollutants^83^.

The SSR is a fascinating topic that is being studied worldwide. However, research results are inconsistent, although some studies suggest the influence of maternal factors and environmental stress on the SSR. There is disagreement among scientists regarding the precise mechanisms operating during this period. It remains uncertain whether it is due to the higher fertilization capacity of sperm carrying the Y chromosome, disparities in the implantation of male and female embryos, or differences arising during embryogenesis^84^. Many scientists believe that under better environmental and maternal conditions, male births are more likely, while under worse conditions, female births are more prevalent. The sex ratio at birth varies by region and country, with more boys than girls reported globally^85^.

In the future, due to Krosno’s proximity to the Polish–Ukrainian border and the war in Ukraine that began in 2022, a potential change in the SSR value after the start of the war would be worth investigating.

### Limitations

The study was conducted on a relatively small group of 11,538 births. However, this size is sufficient to capture the observed trends found in studies with larger datasets. SSR is also influenced by other factors that we did not have in the database. Data regarding the woman's previous pregnancies, sex data of newborns from previous pregnancies, or the number of miscarriages could influence the obtained results.

## Data Availability

Data supporting reported results can be found in https://bip.stat.gov.pl/files/gfx/bip/pl/defaultstronaopisowa/1568/1/1/13._podkarpackie.pdf, https://powietrze.gios.gov.pl/pjp/archives. The data base of mothers and children information have not been made public and are stored by the John Paul II Podkarpackie Province Hospital in Krosno and are available from the corresponding author on reasonable request.
